# The Research Effects of Variable Temperature and Early Strength Agent on the Mechanical Properties of Cement-Stabilized Macadam

**DOI:** 10.3390/ma17153720

**Published:** 2024-07-27

**Authors:** Yanhua Xue, Dongdong Ge, Songtao Lv, Hui Wei, Weiwei Lu, Liangchen Peng

**Affiliations:** 1 National Key Laboratory of Green and Long-Life Road Engineering in Extreme Environment (Changsha), Changsha 410114, China; xueyanhua_2022@stu.csust.edu.cn (Y.X.); lst@csust.edu.cn (S.L.); wh@csust.edu.cn (H.W.); lww_cs@csust.edu.cn (W.L.); plc@stu.csust.edu.cn (L.P.); 2 Xiangjiang Laboratory, Changsha 410114, China

**Keywords:** cement-stabilized macadam, variable temperature curing condition, early strength agent, shrinkage characteristics, fatigue damage characteristics

## Abstract

In cold regions with high daily temperature gradients (>20 °C), the durability of cement-stabilized macadam (CSM) base materials is poor and prone to cracking. To effectively reduce the cracking of semi-rigid base layers in cold regions with high daily temperature gradients and extend fatigue life, this study focused on cracking and fatigue characteristics of CSM with a 10% commercial early strength agent (ESA) added by the external mixing method under different curing conditions. The ESA was manufactured by Jiangsu Subote New Materials Co., Ltd. (Nanjing, China). The curing conditions were divided into variable temperature (0–20 °C) and standard temperature (20 °C). CSM curing was carried out through a programmable curing box. The research results indicated that the variable temperature curing conditions reduced the strength and fatigue resistance of CSM and accelerated the modulus attenuation rate of CSM. At the same time, the drying shrinkage of CSM was greater. The temperature shrinkage coefficient and strain of CSM under variable temperature conditions were smaller than those under standard temperature conditions. The effect of variable temperature conditions on the cracking and durability of CSM could not be ignored in cold regions. Compared to standard temperature curing conditions, the indirect tensile strength of CSM reduced by 31.04% under variable temperature conditions, the coefficient of variation increased by 2.97 times, and the discrete type significantly increased. Compared with CSM without ESA, the dry and temperature shrinkage strains of CSM with 10% ESA were reduced by 24.65% and 26.10%, respectively. At a stress level of 0.6, compared to standard temperature curing conditions, the fatigue life of CSM decreased by 97.19% under variable temperature conditions. Under variable temperature conditions, the fatigue life of CSM with 10% ESA increased by 196 times compared to 0% ESA. Adding ESA enhanced the anti-shrinkage cracking, strength, and durability of CSM under variable temperatures. ESA incorporation effectively compensated for the weakened characteristics of CSM under variable temperature conditions. The study proposed a practical approach for boosting the durability of CSM in cold environments.

## 1. Introduction

Cement-stabilized macadam (CSM) has the benefits of high strength, excellent integrity, good water stability, and frost resistance [1,2,3,4]. As a base material, CSM can decrease the tensile stress at the bottom of the asphalt surface layer. It can also ensure the stability of the roadbed [5]. CSM is a mixture of cement and water added to macadam with a gradation. The rate of cement hydration directly affects the strength of CSM. In low-temperature environments (<20 °C), the hydration reaction rate of cement is slow, which is not conducive to the formation of strength [6,7]. Therefore, the formation of CSM strength is highly susceptible to the influence of environmental temperature [8,9].

Tian et al. [9] observed that when the curing temperature of CSM increased from 5 °C to 10 °C, the 7-day flexural strength increased by 214.3%. When the temperature increased from 10 °C to 20 °C, the 7-day bending tensile strength increased by 157.1%. Soriano et al. and Ryou et al. [10,11,12] pointed out that under low-temperature conditions, an ice interlayer would be generated in the concrete, leading to the fracture of the hydration product gel. At the same time, the freezing of water molecules will generate expansion stress, causing deterioration of the internal interface transition zone of the concrete and loosening of the cement structure, reducing the macroscopic mechanical characteristics of concrete. Ma et al. [13,14,15] observed the effects of different curing temperatures on the mechanical characteristics of CSM, covering unconfined compressive strength, flexural strength, and compressive rebound modulus. This study found that under normal low-temperature conditions, the cement hydration reaction rate of CSM was slow or even stopped, and the durability was poor.

For areas with year-round low temperatures, large temperature differences between day and night, and high water evaporation in cold regions, CSM base materials were more prone to damage [13,16]. During construction in cold regions of China with high daily temperature gradients (>20 °C) in summer, CSM had a fast and large evaporation rate of water, which could lead to drying shrinkage and cracking [17]. The temperature gradient during the curing process could cause temperature stress to exceed the strength formed during its curing period, making it prone to temperature shrinkage and cracking. He et al. [18] conducted a core sample investigation on the road surface of the existing roads in Qinghai Tibet and found that most of the core samples were loose and even showed a high number of non-load-type cracks. Therefore, improving the durability of CSM in cold regions with high daily temperature gradients was of great significance.

Current researchers and scholars have paid much attention to the influence of constant-temperature curing on the characteristics of CSM materials in cold regions with high daily temperature gradients, with the temperature mainly concentrated in the range of −5–20 °C [19]. However, the average temperature in July and August was only 6 °C for the cold regions with high daily temperature gradients, and negative temperatures often occur at night [16]. When the region was in the best construction season, the temperature difference between day and night remained relatively large. Therefore, variable temperature curing of CSM according to the temperature variation in the cold regions with high daily temperature gradients could explore the performance evolution of CSM in harsh environments more realistically [20].

Meanwhile, reducing the cracking of semi-rigid base layers in the cold regions with high daily temperature gradients was crucial for extending the durability of pavements [21,22,23,24]. The study showed that incorporating an early strength agent (ESA) into CSM accelerated the hydration reaction rate of cement, effectively boosted the strength of CSM, and enabled CSM to meet the specification strength requirements at low-temperature conditions. Hang et al. [25,26,27] investigated composite low-temperature ESA’s influence on concrete strength under a 5 °C low-temperature environment. The study found that composite low-temperature ESA significantly raised the early strength of concrete in low-temperature conditions, and the later strength would not decrease. Wang et al. [28] studied the shrinkage characteristics of CSM using ESA. The study found that at the 8% ESA (mixture of single early strength agents Z1, Z2, and Z3 and expansion agent P) dosage, the dry shrinkage coefficient of CSM decreased by 11.2% compared to CSM without adding early strength materials. Tian et al. [29] studied the mechanical, fatigue, and shrinkage characteristics of CSM using ESA. The results shown that ESA significantly boosted the strength, fatigue resistance, and cracking resistance of CSM. Xiong and Lin et al. [30,31,32,33] used different types of ESA to investigate the characteristics enhancement of CSM. Sheng et al. [19] studied the shrinkage characteristics of CSM by combining ESA and brucite fiber.

The incorporation of ESA remarkably boosted the strength, frost resistance, shrinkage cracking, and fatigue characteristics of CSM. However, the existing study focused on the laboratory constant curing temperature, and the effect of ESA on the cracking resistance and fatigue characteristics of CSM under the variable temperature environment in the cold regions with high daily temperature gradients was unclear. Therefore, this study adopted an ESA, based on a variable temperature curing environment, to study the effects of curing conditions on the dry shrinkage, temperature shrinkage, indirect tensile strength, and indirect tensile fatigue behavior of CSM with ESA. The modulus decay equation of CSM was established to study the fatigue damage law of CSM.

## 2. Materials and Methods

### 2.1. Raw Materials

**Figure 1:**
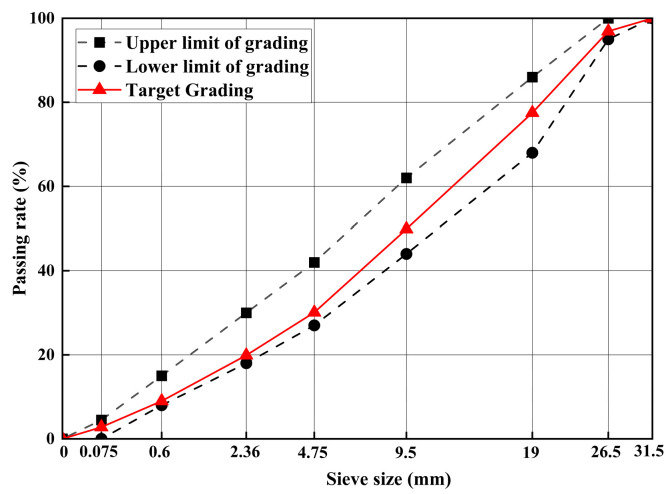
Gradation design of CSM.

According to the basis of previous research [20], this study used PC42.5 cement, and the main technical specifications are illustrated in Table 1. The aggregate type is limestone, and the characteristics indicators are illustrated in Table 2. The ESA is manufactured by Jiangsu Subote New Materials Co., Ltd. (Nanjing, China), and the key technical indexes are illustrated in Table 3. The gradation of CSM used in this study is illustrated in Figure 1.

### 2.2. Preparation of Sample

The specimens were prepared with a cement content of 4.5% [20,35]. The optimal moisture content of CSM obtained through the compaction test was 5.5%, and the maximum dry density was 2.405 g/cm^3^. The dosage of ESA was 10% of the cement dosage [20].

This study used vibration mixing to prepare CSM. The preparation process was as follows: First, the weighed aggregate was added into the mixing pot. Then, it was mixed with water for 90 s in advance, and then cement was incorporated and stirred for 90 s to ensure uniform distribution [20]. Finally, Φ150 mm × 150 mm cylindrical specimens and 400 mm × 100 mm × 100 mm beam specimens were compacted by the vibratory compactor. The specimens were demolded after 12 h to obtain the CSM specimens.

### 2.3. Curing Condition

According to previous research, a programmable curing box was used for variable temperature curing, and the parameters of variable temperature curing are illustrated in Table 4 [20].

The CSM specimens were demolded into plastic bags and placed into a programmable curing chamber. Using the standard temperature curing room as the control group, the temperature in the standard temperature curing room was 20 °C, and the humidity was 95%. The abbreviation of CSM is illustrated in Table 5.

### 2.4. Test Methods

#### 2.4.1. Temperature Shrinkage Test

Based on specification JTG E51-2009 [36], after curing the beam specimen for 7 days and soaking it in water for the last 1 day, the specimen was dried in a 105 °C oven for 10–12 h until it reached a stable weight (quality change less than 0.05%). After measuring the length of the specimen, it was placed on a shrinkage tester and the dial gauge was adjusted. The temperature range of the experiment was −30~60 °C. At the beginning of the experiment, the temperature was raised to 60 °C, and every 10 °C was a stage of gradual cooling, with a cooling rate of 0.5 °C/min. The temperature shrinkage strain and temperature shrinkage coefficient were calculated by Equations (1) and (2).

(1) $$\varepsilon_{i}=\frac{l_{i}-l_{i+1}}{L_{0}}$$

(2) $$\alpha_{i}=\frac{\varepsilon_{i}}{t_{i}-t_{i+1}}$$

In the formula, $\varepsilon_{i}$ represents the temperature shrinkage strain. $\alpha_{i}$ represents the temperature shrinkage coefficient. $l_{i}$ represents the average value (mm) of the sum of the readings on the dial gauge for the *i*-th temperature range. $t_{i}$ represents the *i*-th temperature range set by the temperature control program (°C). $L_{0}$ represents the initial length of the specimen (mm).

#### 2.4.2. Drying Shrinkage Test

Based on specification JTG E51-2009 [36], the beam specimens were cured for 7 days and soaked in water on the last day. After soaking in water, the specimen was dried and the length and mass were measured. The experiment was conducted in a drying shrinkage box with a temperature of 20 ± 1 °C and a humidity of 60 ± 5%. The CSM specimens were divided under each scheme into two groups, with one group of specimens placed on a shrinkage tester to examine for shrinkage deformation. Another group was placed in a drying shrinkage box to examine the drying shrinkage water loss rate. Then, the specimen was placed in a drying chamber and its length and mass were measured daily. After the experiment, the specimen was placed in an oven and heated to a stable weight (quality change less than 0.05%). The water loss rate, shrinkage strain, and dry shrinkage coefficient were calculated by Equations (3)–(7).


(3) $$w_{i}=\left(m_{i}-m_{i+1}\right)/m_{p}$$

(4) $$\delta_{i}=\left(\overset{4}{\underset{j=1}{\sum }}X_{i,j}-\overset{4}{\underset{j=1}{\sum }}X_{i+1,j}\right)/2$$

(5) $$\varepsilon_{i}=\delta_{i}/l$$

(6) $$\alpha_{di}=\varepsilon_{i}/w_{i}$$

(7) $$\alpha_{d}=\frac{\sum \varepsilon_{i}}{\sum w_{i}}$$

In the formula, $w_{i}$ represents the *i*-th water loss rate. $\delta_{i}$ represents the *i*-th drying shrinkage. $\varepsilon_{i}$ represents the *i*-th dry shrinkage strain. $\alpha_{di}$ represents the *i*-th drying shrinkage coefficient. $\alpha_{d}$ represents the total shrinkage coefficient. $m_{i}$ represents the mass of the standard specimen measured for the *i*-th time (g). $X_{i,j}$ represents the reading of the *j*-th dial gauge at the *i*-th test (mm). l represents the length of the standard specimen (mm). $m_{p}$ represents the mass of the standard specimen after drying (g).

#### 2.4.3. Indirect Tensile Strength (ITS) Test

ITS is an essential parameter in the structural design of semi-rigid base materials, which can represent crack resistance under load. According to specification JTG E51-2009 [36], ITS tests were conducted on cylindrical specimens with standard and variable temperature curing for 28 days on a universal testing machine. The loading rate was 1 mm/min. The ITS of the specimen was calculated by Equation (8).

(8) $$R=0.004178\frac{P}{h}$$

In the formula, R represents the ITS of the specimen (Mpa). P represents the maximum pressure at which the specimen fails (N). *h* represents the height of the specimen after immersion in water (mm).

#### 2.4.4. Indirect Tensile Fatigue Test

**Figure 2:**
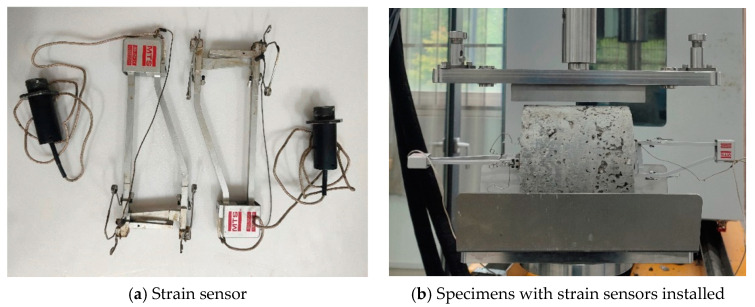
Indirect tensile fatigue test.

The cylindrical specimen was cured for 28 days and the test was conducted after soaking in water for the last 1 day. The surface of the specimen was polished smooth with sandpaper, and two strain sensors were pasted on both sides of the specimen. A strain sensor horizontally measures tensile strain. Another strain sensor measures compressive strain, as illustrated in Figure 2. Indirect tensile fatigue tests were conducted on specimens with strain sensors installed.

#### 2.4.5. Indirect Tensile Modulus

**Figure 3:**
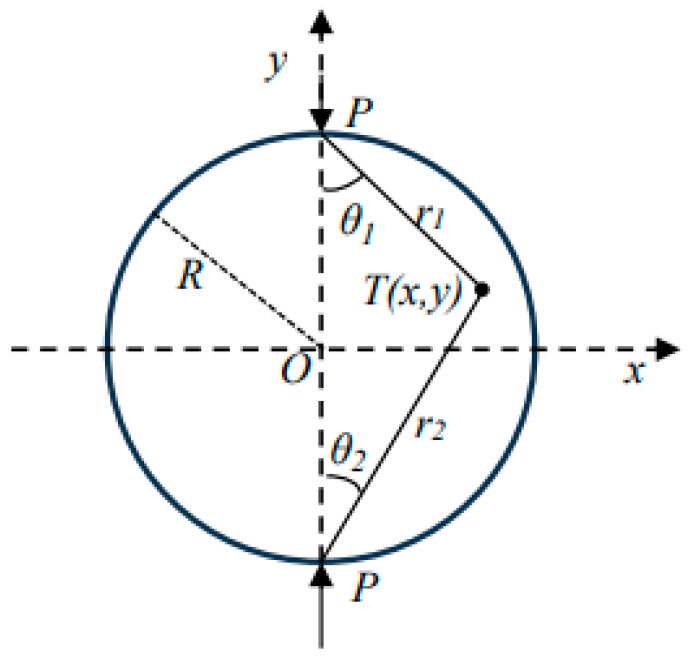
Schematic diagram.

The stress state of the sample in the indirect tensile test is illustrated in Figure 3 [37]. The calculation formula of Brazilian discs was commonly used for indirect tensile tests. According to elasticity, the force distribution at any point T (x, y) inside the disk can be calculated as illustrated in Equation (9).

(9) $$\{\begin{array}{c} \sigma_{x}=\frac{2P}{\pi L}\left(\frac{\sin^{2}\theta_{1}\cos\theta_{1}}{r_{1}}+\frac{\sin^{2}\theta_{2}\cos\theta_{2}}{r_{2}}\right)-\frac{2P}{\pi DL}\\ \sigma_{y}=\frac{2P}{\pi L}\left(\frac{\cos^{3}\theta_{1}}{r_{1}}+\frac{\cos^{3}\theta_{2}}{r_{2}}\right)-\frac{2P}{\pi DL}\\ \sigma_{xy}=\frac{2P}{\pi L}\left(\frac{\sin\theta_{1}\cos^{2}\theta_{1}}{r_{1}}-\frac{\sin\theta_{2}\cos^{2}\theta_{2}}{r_{2}}\right) \end{array}$$

In the formula, *P* is the load (N), *L* is the length (thickness) of the cylindrical specimen (mm), and *D* is the diameter of the cylindrical specimen (mm).

Chen et al. [38,39] derived the tensile and compressive modulus equations in indirect tensile fatigue testing based on the strain gauge testing method, as illustrated in Equation (10).

(10) $$\{\begin{array}{c} E_{x}=\frac{4P}{\pi L}\times \frac{ab+cd\mu^{2}}{b\Delta \mu -\mu d\Delta v}\\ E_{y}=\frac{4P}{\pi L}\times \frac{ab+cd\mu^{2}}{\mu c\Delta \mu +a\Delta v} \end{array}$$

Among them:

(11) $$\{\begin{array}{c} a=\frac{Dl}{D^{2}+l^{2}}-\arctan\frac{l}{D}+\frac{l}{2D}\\ b=\frac{l}{2D}-\ln\frac{D-l}{D+l}\\ c=\frac{l}{2D};\Delta \mu =\varepsilon_{\mu }l;\Delta v=\varepsilon_{v}l\\ d=\frac{Dl}{D^{2}+l^{2}}+\arctan\frac{l}{D}-\frac{l}{2D} \end{array}$$

$\varepsilon_{\mu }$ and $\varepsilon_{v}$ represent the horizontal and vertical strains of the specimen tested by the strain sensor. l is the initial distance between the two ends of the strain sensor (50 mm).

## 3. Shrinkage Test Results and Discussion

### 3.1. Analysis of Temperature Shrinkage Results

**Figure 4:**
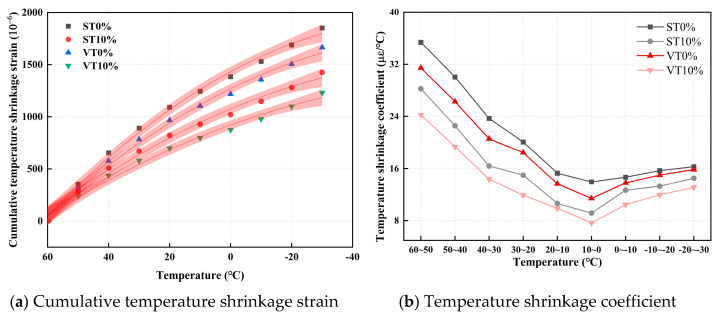
Temperature shrinkage results of CSM.

The confidence intervals for the temperature shrinkage strains and the temperature shrinkage coefficients are displayed in Figure 4.

As illustrated in Figure 4a, the cumulative temperature shrinkage strain of ST0%, ST10%, VT0%, and VT10% continues to increase with the temperature decrease. The cumulative temperature shrinkage strains of ST0%, ST10%, VT0%, and VT10% are 1851.06 με, 1425.55 με, 1664.76 με, and 1230.18 με, respectively. Adding ESA can reduce the temperature shrinkage strain of CSM. Incorporating 10% ESA reduces the temperature shrinkage strain by 22.99% and 26.10% in standard and variable temperature curing environments, respectively. Under variable temperature curing conditions, the temperature shrinkage strain of CSM was less. Compared with ST0%, the temperature shrinkage strain of VT0% was reduced by 10.06%.

As illustrated in Figure 4b, the temperature shrinkage coefficients of ST0%, ST10%, VT0%, and VT10% show a trend of first reducing and then growing with the decline in temperature and reaching the lowest point within the range of 0–10 °C. The reason may be that as the temperature decreases, the thermal motion of pore water molecules in CSM weakens, the molecular spacing decreases, the attraction increases, and the surface tension increases. This effect leads to specimen volume shrinkage. The surface tension in the early cooling stage was high, causing significant temperature shrinkage deformation and temperature shrinkage coefficient reduction. The surface tension reduces as the temperature declines, and the temperature shrinkage coefficient gradually reduces. When the temperature is less than 0 °C, the pore water freezes, causing volume expansion and offsetting some shrinkage deformation, increasing the temperature shrinkage coefficient [40]. Under variable temperature curing conditions, compared with CSM with 0% ESA, CSM with 10% ESA showed a 22.94% decrease in the temperature shrinkage coefficient at 50–60 °C. At 50–40 °C, it decreased by 24.46%. At 40–30 °C, it reduced by 25.94%. At 30–20 °C, it reduced by 27.75%. At 20–10 °C, it reduced by 27.71%. At 10–0 °C, it reduced by 28.21%. At −10–0 °C, it reduced by 27.79%. At −20–−10 °C, it decreased by 27.04%. At −30–−20 °C, it decreased by 26.10%. As the temperature gradually decreases from high temperature, the effect of ESA on the temperature shrinkage coefficient of CSM shows a trend of first increasing and then decreasing. ESA has the greatest impact within the temperature range of 0–20 °C, and the decrease in temperature shrinkage coefficient is the most significant.

### 3.2. Analysis of Drying Shrinkage Test Results

**Figure 5:**
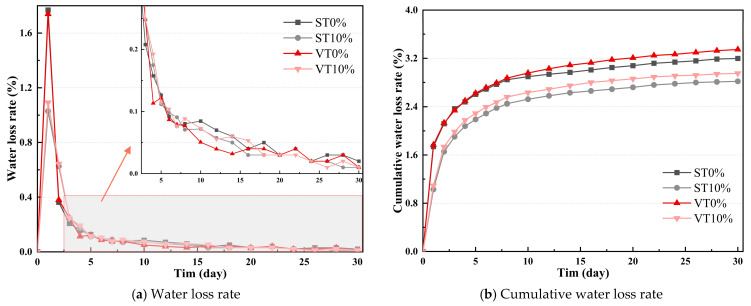
Water loss rate of CSM.

**Figure 6:**
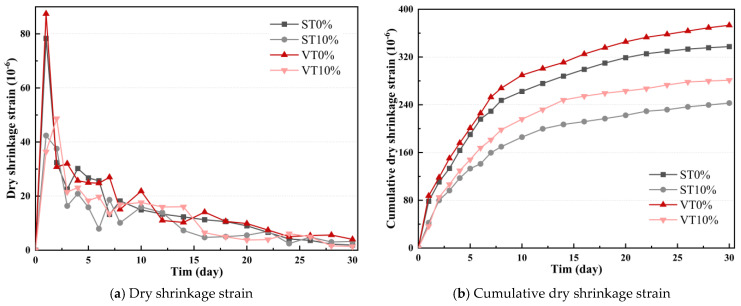
Dry shrinkage strain test results of CSM.

The changes in water loss rate, dry shrinkage strain, and dry shrinkage coefficient with time of CSM are illustrated in Figure 5 and Figure 6. Statistical analysis of the water loss rate is illustrated in Table 6.

As illustrated in Figure 5, the water loss rate of CSM showed an increase and then a fall with time and gradually tended to stabilize. The cumulative water loss rate of CSM rose continuously with time and gradually stabilized in the later stage of the experiment. The main reason was that in the early stage of the drying shrinkage test, the water lost was mainly free water filled in the pores of CSM, which is abundant and easy to evaporate, so the water loss rate increased rapidly. In the later stage of the experiment, the internal moisture of CSM was mainly adsorbed water and interlayer water, which were difficult to evaporate. Therefore, the water loss rate of CSM would decrease and tend to stabilize.

After adding ESA, the water loss rate of CSM significantly decreased. The cumulative water loss rates of ST0% and VT0% were greater than ST10% and VT10%, respectively. This may be because ESA accelerated cement hydration, and consuming free water and some capillary water in CSM reduces the water loss rate of ST10% and VT10%. The cumulative water loss rates of VT0% and VT10% were higher than ST0% and ST10%, respectively. It may be that the cement hydrates slower and consumes less water under variable temperature conditions. More water was evaporated in the dry shrinkage test session. The statistical results of the water loss rate show that the discreteness of the drying shrinkage test of CSM under different health conditions is relatively small.

As illustrated in Figure 6, the variation in drying shrinkage strain of CSM with time was consistent with the law of water loss rate. In the early stage of the experiment, the drying shrinkage strain was relatively large, and in the later stage, the shrinkage strain gradually stabilized. After 30 days of shrinkage, the cumulative shrinkage strains of ST0%, ST10%, VT0%, and VT10% were 373.15 με, 242.87 με, 337.46 με, and 281.17 με, respectively. Compared to ST0% and VT0%, the cumulative shrinkage strain of ST10% and VT10% decreased by 28.03% and 24.65%, respectively. Adding ESA can diminish the dry shrinkage strain of CSM [41].

Compared to ST0% and ST10%, the cumulative shrinkage strain of VT0% and VT10% increased by 10.58% and 15.77%, respectively. Under variable temperature conditions, the drying shrinkage of CSM was greater. This may be because the variable temperature conditions inhibit cement hydration, produce fewer hydration products, and consume less water, so the faster the water evaporates in the test stage, the greater the drying shrinkage deformation. Related studies have also confirmed that the shrinkage deformation of CSM was greater under low-temperature conditions [19], and adding ESA can enhance the shrinkage characteristics of the CSM under low temperature.

**Figure 7:**
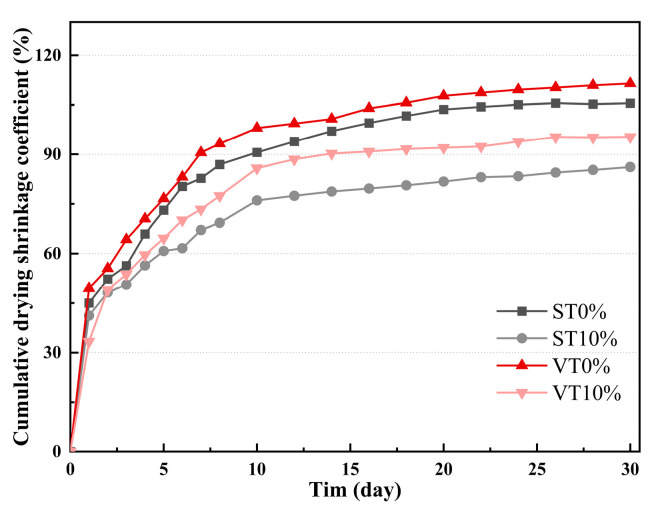
Cumulative drying shrinkage coefficient of CSM.

Figure 7 illustrates that the cumulative drying shrinkage coefficients of ST0%, ST10%, VT0%, and VT10% were 105.52%, 86.16%, 111.49%, and 95.30%, respectively. Compared with ST0% and VT0%, the shrinkage coefficients of ST10% and VT10% were reduced by 19.36% and 16.19%. This indicates that ESA significantly impacts the shrinkage coefficient of CSM under different temperatures.

## 4. Results and Discussion of Strength and Fatigue Tests

### 4.1. ITS Analysis

The results of ITS of CSM are illustrated in Table 7.

As illustrated in Table 6, the ITS values of ST0% and ST10% were significantly higher than those of VT0% and VT10%. The variable temperature curing environment was not conducive to forming CSM strength. Compared to ST0%, the ITS of ST10% increased by 43.38%. Compared to VT0%, the ITS of VT10% increased by 60.36%. The incorporation of ESA raised the ITS of CSM in different curing conditions. The coefficient of variation results shown that the intensity variability of VT0% was the highest. The coefficient of variation increased by 2.97 times. The performance of CSM was more discrete in variable temperature curing environments. After adding ESA under variable temperature conditions, the coefficient of variation of the ITS results of CSM decreased, indicating that the addition of ESA can effectively improve the discrete strength of CSM under variable temperature conditions.

### 4.2. Fatigue Characteristics

**Figure 8:**
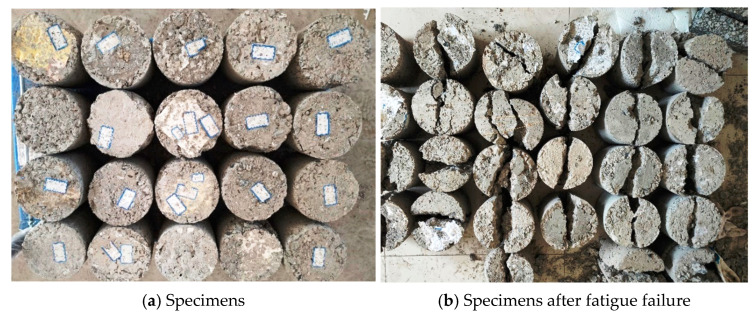
Fatigue test.

Different stress levels are selected for fatigue testing based on the ITS results. The fatigue specimen is illustrated in Figure 8. The fatigue results are illustrated in Table 8.

As illustrated in Table 7, the fatigue life of CSM diminishes continuously with the increase in stress level. When the stress level is 0.6 MPa, the fatigue lives of ST0%, ST10%, VT0%, and VT10% were 4704, 181,616, 132, and 25,916, respectively. The fatigue life of ST0% was 35 times that of VT0%. The fatigue life of ST10% was seven times that of VT10%. The fatigue life of CSM was significantly reduced under a variable temperature curing environment. Adding ESA raised the durability of CSM under variable temperature conditions.

**Figure 9:**
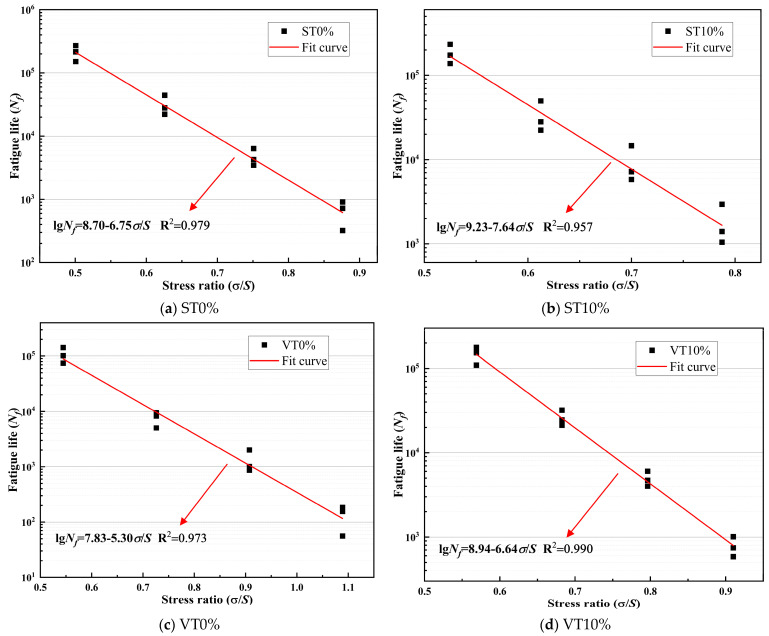
Fatigue curves of CSM with different stress ratios.

**Figure 10:**
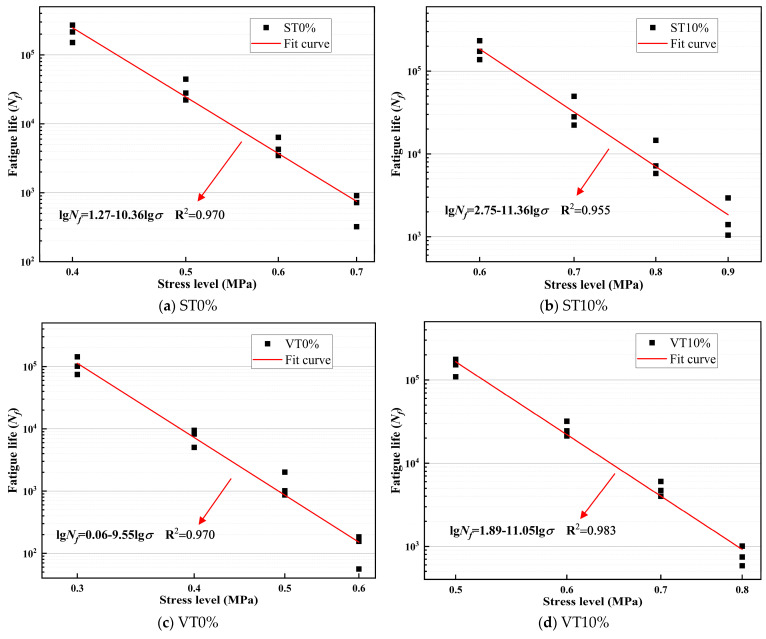
Fatigue curves of CSM at different stress levels.

According to the fatigue test results, fatigue curve fitting was performed on CSM with different stress levels and stress ratios. The fitting results are illustrated in Figure 9 and Figure 10. The fatigue equation and related parameters are illustrated in Table 9 and Table 10.

Parameters *a* and *b* can reflect the fatigue characteristics of CSM. Among them, the larger the value of *a*, the superior the fatigue resistance of CSM. The larger the value of *b*, the more significant the impact of stress changes on the fatigue life of CSM.

The fatigue life of CSM has a very strong correlation with stress levels and stress ratios [42]. Compared to the stress level, the R^2^ in the fatigue equation of CSM based on the stress ratio was higher. The degree of fatigue equation fitting for CSM based on the stress ratio was better. The *a*-values order for CSM under different stress levels or ratios was ST10% > VT10% > ST0% > VT0%. Among them, VT0% has the smallest *a*-value.

Under the same curing conditions, the fatigue life of CSM with 10% ESA added was significantly larger than that of CSM without ESA added, and the coefficient *a* was also significantly increased. ESA greatly boosted the fatigue life of CSM. This was primarily because the incorporation of ESA significantly accelerated the cement hydration reaction rate and the strength formation rate of CSM, thereby improving the fatigue resistance of the CSM. On the other hand, the fatigue life and coefficient *a*-value of the specimens under standard temperature environments are greater than those under variable temperature environments. The durability of CSM under variable temperature was inferior to that of standard temperature, and the incorporation of ESA greatly boosted the fatigue resistance of CSM in variable temperature conditions.

### 4.3. Test Methods

A modulus value can be obtained for each load cycle when conducting indirect tensile fatigue tests. Due to the large amount of fatigue test data, this study used Python software programming to process the data, calculate the modulus, and analyze the modulus decay of CSM.

#### 4.3.1. Determination of the Indirect Tensile Initial Modulus Value

The indirect tensile initial modulus (E0) of CSM specimens was usually taken as the modulus (MPa) after 50 loading cycles. However, considering that the fatigue life of CSM varies greatly at different stress levels, this method was prone to deviation. This study selected the average value of 10 moduli near $N/N_{f}=0.01$ as the indirect tensile initial modulus to improve the accuracy of the data. Table 11, Table 12, Table 13 and Table 14 summarize the initial values of CSM.

As illustrated in Table 11, Table 12, Table 13 and Table 14, the coefficient of variation of the indirect tensile initial modulus of CSM ranges from 3.41% to 11.97%, with relatively small variability. The indirect tensile initial modulus of CSM rises with the increase in stress level. The modulus of CSM was significantly affected by different curing environments and ESA content. The initial modulus of ST0% ranges from 26,900 to 35,800 MPa. The indirect tensile initial modulus of ST10% ranges from 32,200 to 46,700 MPa. The indirect tensile initial modulus of VT0% was between 10,800 and 20,600 MPa; the initial modulus of VT10% was between 30,800–42,600 MPa. At a stress level of 0.6 MPa, the average initial modulus values of ST0%, ST10%, VT0%, and VT10% were 32,383 MPa, 35,241 MPa, 20,529 MPa, and 34,413 MPa, respectively. It can be seen that variable temperature curing conditions have an adverse effect on the dynamic modulus of CSM. After adding 10% ESA, the stiffness of CSM significantly improved.

#### 4.3.2. Determination of the Indirect Tensile Critical Modulus Value

**Figure 11:**
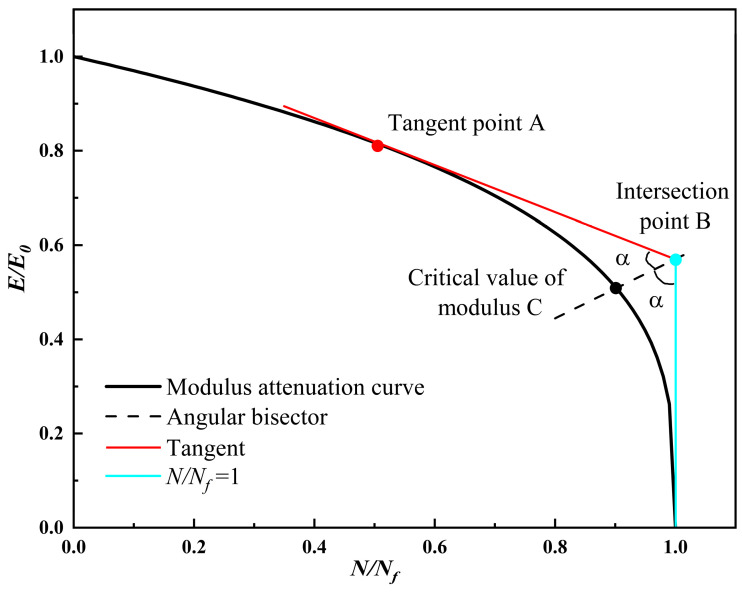
Schematic diagram of modulus critical point.

In fatigue testing, the modulus of the specimen will gradually decrease with the application of cyclic load. The average modulus of the first five cycles before the end of fatigue testing was mainly used as the critical modulus value ($E_{min}$). CSM is a brittle material whose modulus was significantly affected by stress and was prone to fracture under high stress. The average modulus of the five cycles before the end of the fatigue test was taken as the critical value, and there was a high degree of variability. To reduce the discreteness of experimental data, the tangent line to the point corresponding to $N/N_{f}=0.5$ on the modulus decay curve and the straight line at $N/N_{f}=1$ were utilized, and the intersection of the angular bisector of the intersection of the two lines and the modulus decay curve was chosen to be the fatigue modulus critical value [43], which is illustrated in Figure 11.

In the formula, E_0_ is the initial modulus of the specimen, and E is the remaining modulus of the specimen after the load was applied. Point A is the point corresponding to the tangent on the fatigue modulus decay curve. Point B is the intersection point between the tangent of the point corresponding to $N/N_{f}$ = 0.5 on the fatigue modulus decay curve and the straight line at $N/N_{f}$ = 1. α the bisector angle between two intersecting lines.

The critical modulus values of CSM at different stress levels are illustrated in Table 15, Table 16, Table 17 and Table 18.

As illustrated in Table 15, Table 16, Table 17 and Table 18, the coefficient of variation of the critical modulus of CSM ranges from 0.77% to 16.49%, with relatively small variability. The critical modulus value of CSM reduces as the stress level grows. The modulus of CSM was significantly affected by environments and ESA content. The critical modulus value of ST0% was between 9500 and 13,600 MPa, ST10% was between 9400 and 21,000 MPa, VT0% was between 3400 and 6000 MPa, and VT10% was between 10,000 and 18,000 MPa. At a stress level of 0.6 MPa, the average critical values of modulus for ST0%, ST10%, VT0%, and VT10% are 11,230 MPa, 21,094 MPa, 3418 MPa, and 15,636 MPa, respectively. Under the same dosage of ESA, the critical value of the modulus of CSM in a variable temperature curing environment was lower than that in a standard temperature curing environment. After adding 10% ESA, the critical modulus values of the CSM under different curing conditions all increased.

#### 4.3.3. Analysis of Fitting Results for Modulus Decay of CSM

**Figure 12:**
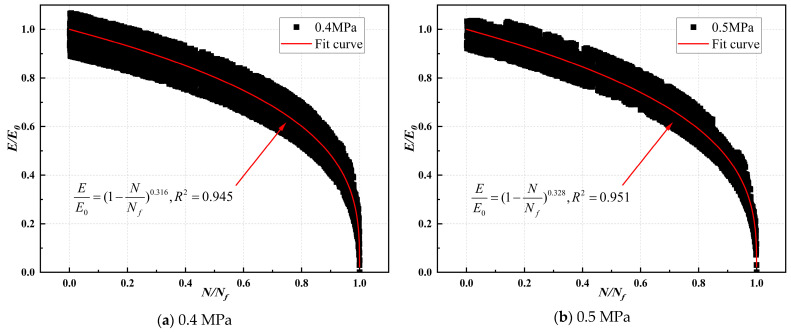

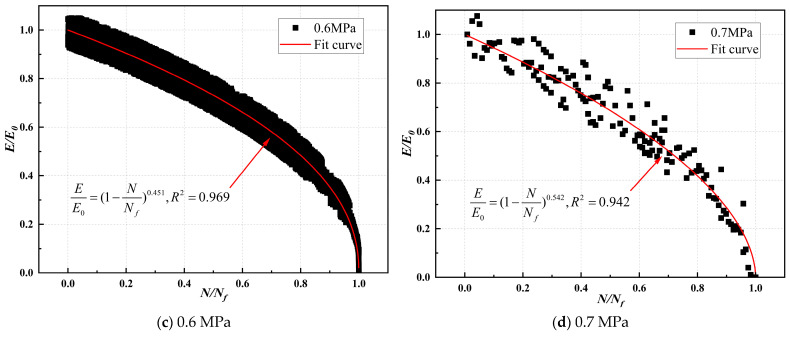
Modulus decay of ST0% under different stress levels.

**Figure 13:**
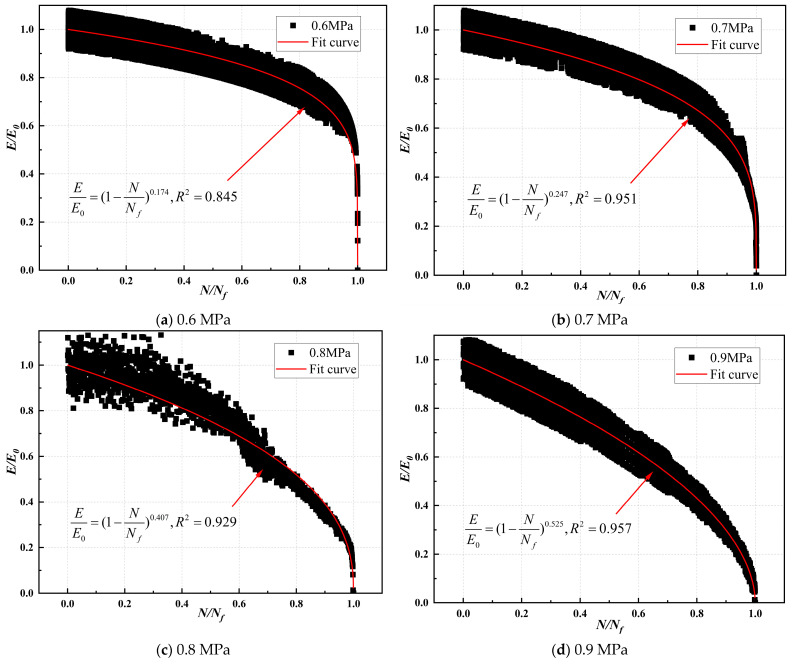
Modulus decay of ST10% under different stress levels.

**Figure 14:**
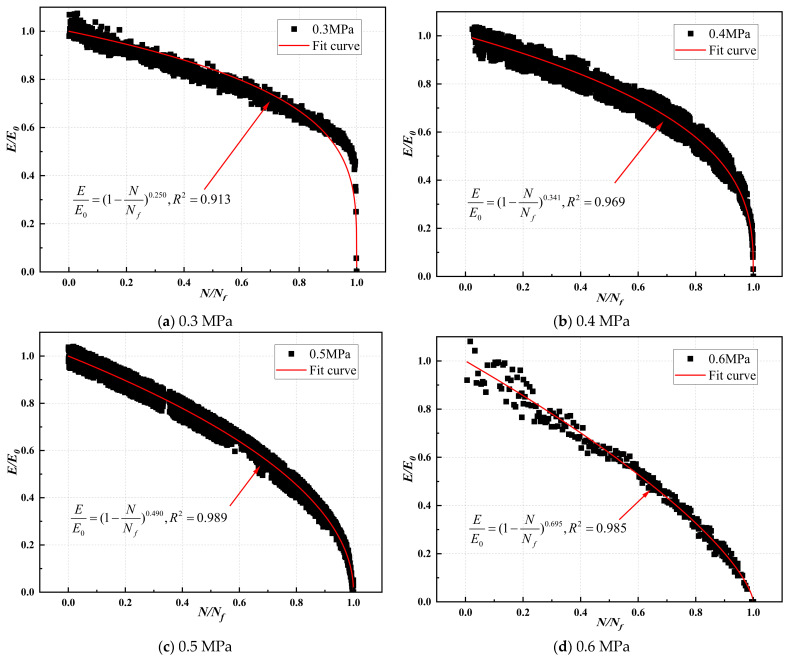
Modulus decay of VT0% under different stress levels.

**Figure 15:**
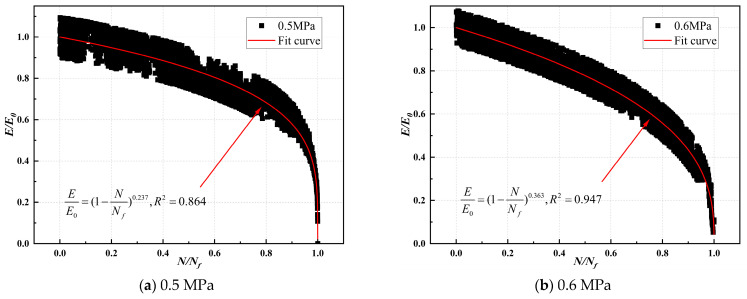

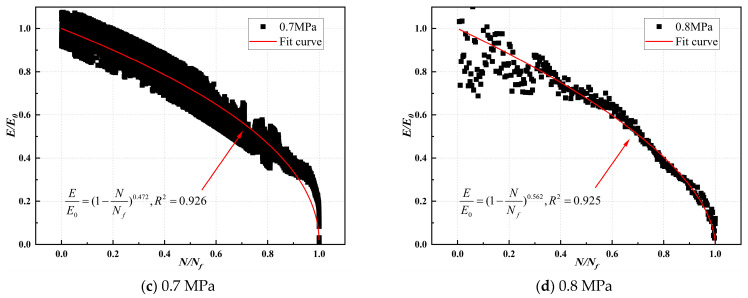
Modulus decay of VT10% under different stress levels.

The compression modulus ratio and fatigue life ratio of CSM were fitted, and the modulus decay model parameters are illustrated in Table 19, Table 20, Table 21 and Table 22. The modulus decay of ST0%, ST10%, VT0%, and VT10% is illustrated in Figure 12, Figure 13, Figure 14 and Figure 15.

Table 19, Table 20, Table 21 and Table 22 show that the modulus decay curves of CSM were similar at different stress levels. Under load, the internal structure of the specimen was gradually damaged, and the modulus gradually decreased until it failed. This indicated that the damage of CSM was a non-linear accumulation process. However, the curing temperature and ESA affected the internal damage process of CSM. The larger the value of *m*, the faster the modulus attenuation, indicating more internal damage to CSM under the same load. Table 19, Table 20, Table 21 and Table 22 show that the rate of CSM modulus attenuation was related to the stress level. As the stress level increased, the CSM modulus decay fitting parameter *m*-value gradually increased, and the CSM modulus decay rate increased.

At a stress level of 0.6 MPa, the modulus decay curve parameters m for ST0%, ST10%, VT0%, and VT10% were 0.453, 0.179, 0.682, and 0.361, respectively. Under the same curing conditions, ST0% and VT0% values were significantly higher than those of ST10% and VT10%. CSM without ESA addition had greater damage under load and a faster performance degradation rate. Adding 10% ESA to CSM improved its fatigue resistance. The *m*-value of CSM under variable temperature was lower than that under standard temperatures. The primary reason was that under variable temperature conditions, the cement hydration rate in CSM was slow, the strength formation of CSM was slow, the adhesion between colloids was poor, and it was easy to cause damage under load.

## 5. Conclusions

This study investigated the influence of temperature on temperature shrinkage, drying shrinkage, indirect tensile strength, fatigue, and modulus characteristics of CSM with ESA. The conclusions obtained were as follows:

(1) The variable temperature curing conditions adversely affected the drying shrinkage characteristics of CSM. After adding 10% ESA, the drying shrinkage coefficient of CSM decreased by 16.19%. Adding ESA effectively accelerates cement hydration, consumes free water and capillary water in CSM, and effectively improves the anti-shrinkage cracking performance of CSM. However, compared to ST0%, the temperature shrinkage coefficient of VT0% decreased by 10.06%. The variable temperature curing conditions improved the temperature shrinkage characteristics of CSM;
(2) Compared with a standard temperature curing environment, under a variable temperature curing environment, the strength and fatigue life at 0.6 MPa of CSM decreased by 31.04% and 97.19%, respectively. The modulus decay curve parameters at 0.6 MPa increased by 51.21%. The variable temperature curing environment weakened the strength and fatigue resistance of CSM and accelerated the rate of modulus decay. The addition of ESA significantly boosted the durability of CSM under variable temperature conditions;
(3) The variable temperature service environment and the addition of ESA will significantly affect the durability of CSM. In cold regions with high daily temperature gradients, it is necessary to consider the effect of variable temperature conditions on the characteristics of CSM;
(4) The incorporation of ESA compensates for the weakened characteristics of CSM under variable temperature conditions and is a practical approach to boost CSM characteristics in cold regions with high daily temperature gradients (>20 °C).

This study only focuses on specific ESA and variable temperature conditions. In the future, more types of ESA and more severe service conditions can be considered to affect the characteristics of CSM.

## Figures and Tables

**Table 1:** Technical indexes of cement.

Property		Results	Requirement	Standard Deviation
Setting time	Initial setting time/min	270	≥180	9.3
	Final setting time/min	395	≥360, ≤180	12.1
3D compressive strength/MPa		23.8	≥3.5	3.69
3D flexural strength/MPa		4.9	≥17	1.45

**Table 2:** Limestone characteristics indicators results.

Property	Requirement	Coarse Aggregate Test Results				Standard Deviation	Test Method
		19~ 31.5 mm	9.5~ 19.5 mm	4.75~ 9.5 mm	0~ 4.75 mm		
Crushing value/%	≤26%	21.3	18.9	-	-	3.61	T 0316-2005 [34]
Gross volume relative density/(g/cm^3^)	Actual measurement records	2.230	2.730	2.607	2.589	0.59	T 0316-2005 [34]
Apparent density/(g/cm^3^)	Actual measurement records	2.821	2.763	2.731	2.692	0.93	T 0312-2005 [34]
Water absorption rate/%	Actual measurement records	0.4	0.6	1.3	-	0.69	T 0312-2005 [34]
Liquid limit/plasticity index of particles less than 0.6 mm	Liquid limit ≤ 28%	23%				2.46	T 0118-2005 [34]
	Plasticity index ≤ 9	3.2				1.03	T 0118-2005 [34]

**Table 3:** Characteristics indexes of ESA.

Property	Test Results	Standard Deviation
Appearance status	Powder	-
1.18 mm sieve residue (%)	≤0.05	0.014
Setting time difference (min)	≤30	2.45
24 h cement paste strength ratio (%)	≥125	16.16

**Table 4:** Variable temperature curing parameters.

Indicator	Ranges					
Temperature	15~20 °C	20~15 °C	15~5 °C	5~0 °C	0~5 °C	5~15 °C
Duration	4 h	4 h	4 h	6 h	3 h	3 h

**Table 5:** Abbreviation of CSM.

Curing Condition	ESA Content	
	0%	10%
Standard temperature curing	ST0%	ST10%
Variable temperature curing	VT0%	VT10%

**Table 6:** Statistical analysis of water loss rate.

Time (Day)	ST0%		ST10%		VT0%		VT10%	
	Average Value	Variance	Average Value	Variance	Average Value	Variance	Average Value	Variance
0	0	0	0	0	0	0	0	0
2	0.36	0.000107	0.63	0.000184	0.38	0.000181	0.64	0.000186
4	0.15	0.000059	0.17	0.000037	0.11	0.000065	0.19	0.000094
6	0.09	0.000036	0.10	0.000064	0.09	0.000071	0.10	0.000039
8	0.08	0.000068	0.07	0.000058	0.08	0.000091	0.09	0.000058
10	0.08	0.000072	0.07	0.000063	0.05	0.000064	0.07	0.000064
12	0.07	0.000091	0.06	0.000024	0.04	0.000082	0.06	0.000039
14	0.06	0.000083	0.05	0.000081	0.03	0.000047	0.06	0.000031
16	0.04	0.000101	0.03	0.000047	0.04	0.000059	0.05	0.000064
18	0.05	0.000076	0.03	0.000039	0.04	0.000038	0.03	0.000061
20	0.03	0.000058	0.03	0.000057	0.03	0.000021	0.03	0.000038
22	0.04	0.000069	0.04	0.000069	0.04	0.000019	0.03	0.000075
24	0.02	0.000066	0.02	0.000076	0.02	0.000033	0.02	0.000060
26	0.03	0.000090	0.02	0.000073	0.02	0.000031	0.01	0.000035
28	0.03	0.000046	0.01	0.000042	0.03	0.000067	0.02	0.000066
30	0.02	0.000052	0.01	0.000034	0.01	0.000051	0.01	0.000055

**Table 7:** ITS test results.

	ITS (MPa)			Average Value (MPa)	Coefficient of Variation (%)
	1	2	3		
ST0%	0.837	0.761	0.799	0.799	3.85
ST10%	1.042	1.164	1.224	1.143	6.62
VT0%	0.554	0.472	0.627	0.551	11.45
VT10%	0.883	0.947	0.808	0.879	6.46

**Table 8:** Fatigue test results.

	Indirect Tensile Strength (MPa)	Stress Level (MPa)	Stress Ratio	Fatigue Life			Average Value
				1	2	3	
ST0%	0.799	0.4	0.50	151,407	270,604	215,586	212,532
		0.5	0.63	27,969	44,512	22,217	31,566
		0.6	0.75	6368	4274	3469	4704
		0.7	0.88	908	322	721	650
ST10%	1.143	0.6	0.52	173,616	233,324	137,908	181,616
		0.7	0.61	28,124	49,702	22,339	33,388
		0.8	0.70	5811	7181	14616	9203
		0.9	0.79	1046	1403	2948	1799
VT0%	0.551	0.3	0.54	142,927	74,708	101,628	106,421
		0.4	0.73	8269	9475	5038	7594
		0.5	0.91	2019	1014	867	1300
		0.6	1.09	184	56	156	132
VT10%	0.879	0.5	0.57	152,807	177,146	109,533	146,495
		0.6	0.68	21,252	24,526	31,969	25,916
		0.7	0.80	4009	6040	4710	4920
		0.8	0.91	745	585	1010	780

**Table 9:** Fatigue equation parameters of CSM under different stress ratios.

	*a*	*b*	Fatigue Equation	R^2^
ST0%	8.70	−6.75	${\mathrm{lgN}}_{f}=8.70-6.75\sigma$/S	0.979
ST10%	9.23	−7.64	${\mathrm{lgN}}_{f}=9.23-7.64\sigma /S$	0.957
VT0%	7.83	−5.30	${\mathrm{lgN}}_{f}=7.83-5.30\sigma /S$	0.973
VT10%	8.94	−6.64	${\mathrm{lgN}}_{f}=8.94-6.64\sigma /S$	0.990

**Table 10:** Fatigue equation parameters of CSM under different stress levels.

	*a*	*b*	Fatigue Equation	R^2^
ST0%	1.27	−10.36	${\mathrm{lgN}}_{f}=1.27-10.36\mathrm{lg}\sigma$	0.970
ST10%	2.72	−11.36	${\mathrm{lgN}}_{f}=2.75-11.36\mathrm{lg}\sigma$	0.955
VT0%	0.06	−9.55	${\mathrm{lgN}}_{f}=0.06-9.550\mathrm{lg}\sigma$	0.970
VT10%	1.89	−11.05	${\mathrm{lgN}}_{f}=1.89-11.05\mathrm{lg}\sigma$	0.983

**Table 11:** Initial values of modulus for ST0%.

Stress Level (MPa)	Number	*E*_0_ (MPa)	Average Value (MPa)	Coefficient of Variation (%)
0.4	1	27,365	26,920	4.23
	2	28,037		
	3	25,358		
0.5	1	30,777	30,557	2.81
	2	29,414		
	3	31,479		
0.6	1	32,105	32,383	5.41
	2	30,390		
	3	34,655		
0.7	1	39,442	35,755	7.40
	2	34,460		
	3	33,362		

**Table 12:** Initial values of modulus for ST10%.

Stress Level (MPa)	Number	*E*_0_ (MPa)	Average Value (MPa)	Coefficient of Variation (%)
0.6	1	33,599	35,241	4.67
	2	34,635		
	3	37,489		
0.7	1	38,445	39,850	3.41
	2	39,417		
	3	41,689		
0.8	1	43,106	44,411	7.98
	2	49,252		
	3	40,875		
0.9	1	51,956	46,680	8.82
	2	41,912		
	3	46,173		

**Table 13:** Initial values of modulus for VT0%.

Stress Level (MPa)	Number	*E*_0_ (MPa)	Average Value (MPa)	Coefficient of Variation (%)
0.3	1	12,384	10,855	10.54
	2	10,547		
	3	9634		
0.4	1	12,482	12,578	11.97
	2	14,467		
	3	10,784		
0.5	1	16,258	16,158	6.36
	2	17,364		
	3	14,852		
0.6	1	18,344	20,529	8.24
	2	22,463		
	3	20,779		

**Table 14:** Initial values of modulus for VT10%.

Stress Level (MPa)	Number	*E*_0_ (MPa)	Average Value (MPa)	Coefficient of Variation (%)
0.5	1	33,162	30,895	6.91
	2	28,036		
	3	31,486		
0.6	1	33,193	34,413	3.29
	2	35,921		
	3	34,125		
0.7	1	39,048	37,349	5.28
	2	38,414		
	3	34,585		
0.8	1	41,302	45,221	5.72
	2	45,151		
	3	49,210		

**Table 15:** Critical values of modulus for ST0%.

Stress Level (MPa)	Number	*E_min_* (MPa)	Average Value (MPa)	Coefficient of Variation (%)
0.4	1	13,235	13,536	4.99
	2	14,472		
	3	12,901		
0.5	1	14,534	14,648	3.13
	2	15,258		
	3	14,151		
0.6	1	11,174	11,230	11.97
	2	9612		
	3	12,905		
0.7	1	10,591	9585	9.31
	2	8422		
	3	9741		

**Table 16:** Critical values of modulus for ST10%.

Stress Level (MPa)	Number	*E_min_* (MPa)	Average Value (MPa)	Coefficient of Variation (%)
0.6	1	21,581	21,094	4.99
	2	21,424		
	3	20,278		
0.7	1	21,773	20,331	3.13
	2	21,794		
	3	17,427		
0.8	1	16,817	17,060	11.97
	2	17,142		
	3	17,221		
0.9	1	14,764	9418	9.31
	2	7482		
	3	6008		

**Table 17:** Critical values of modulus for VT0%.

Stress Level (MPa)	Number	*E_min_* (MPa)	Average Value (MPa)	Coefficient of Variation (%)
0.3	1	6931	6017	10.81
	2	5469		
	3	5652		
0.4	1	5665	5575	6.00
	2	5933		
	3	5128		
0.5	1	5085	5140	0.77
	2	5174		
	3	5162		
0.6	1	2853	3418	20.61
	2	2989		
	3	4411		

**Table 18:** Critical values of modulus for VT10%.

Stress Level (MPa)	Number	*E_min_* (MPa)	Average Value (MPa)	Coefficient of Variation (%)
0.5	1	18,746	18,098	2.57
	2	17,678		
	3	17,871		
0.6	1	14,432	15,636	8.19
	2	15,067		
	3	17,410		
0.7	1	12,182	13,232	10.82
	2	12,258		
	3	15,256		
0.8	1	10,487	10,589	16.49
	2	8503		
	3	12,777		

**Table 19:** Modulus decay model parameters for ST0%.

Stress Level (MPa)	Stress Ratio (*t*)	Number	*m*-Value	R^2^	*m*-Average Value	*m*-Standard Deviation	Coefficient of Variation (%)
0.4	0.501	1	0.316	0.945	0.299	0.012	4.11
		2	0.289	0.921			
		3	0.291	0.955			
0.5	0.626	1	0.328	0.951	0.321	0.026	8.01
		2	0.287	0.943			
		3	0.349	0.927			
0.6	0.751	1	0.451	0.969	0.453	0.026	5.69
		2	0.486	0.954			
		3	0.423	0.939			
0.7	0.876	1	0.542	0.942	0.543	0.024	4.36
		2	0.572	0.919			
		3	0.514	0.901			

**Table 20:** Modulus decay model parameters for ST10%.

Stress Level (MPa)	Stress Ratio (*t*)	Number	*m*-Value	R^2^	*m*-Average Value	*m*-Standard Deviation	Coefficient of Variation (%)
0.6	0.525	1	0.174	0.845	0.179	0.021	11.57
		2	0.206	0.914			
		3	0.156	0.892			
0.7	0.612	1	0.247	0.951	0.269	0.023	8.67
		2	0.258	0.946			
		3	0.301	0.936			
0.8	0.700	1	0.407	0.929	0.411	0.031	7.48
		2	0.451	0.903			
		3	0.376	0.947			
0.9	0.787	1	0.525	0.957	0.640	0.087	13.57
		2	0.661	0.938			
		3	0.735	0.952			

**Table 21:** Modulus decay model parameters for VT0%.

Stress Level (MPa)	Stress Ratio (*t*)	Number	*m*-Value	R^2^	*m*-Average Value	*m*-Standard Deviation	Coefficient of Variation (%)
0.3	0.544	1	0.250	0.913	0.256	0.023	9.08
		2	0.287	0.907			
		3	0.231	0.871			
0.4	0.726	1	0.341	0.969	0.351	0.026	7.49
		2	0.387	0.948			
		3	0.325	0.930			
0.5	0.907	1	0.490	0.989	0.483	0.023	4.66
		2	0.507	0.934			
		3	0.453	0.916			
0.6	1.089	1	0.695	0.985	0.682	0.051	7.53
		2	0.738	0.920			
		3	0.614	0.917			

**Table 22:** Modulus decay model parameters for VT10%.

Stress Level (MPa)	Stress Ratio (*t*)	Number	*m*-Value	R^2^	*m*-Average Value	*m*-Standard Deviation	Coefficient of Variation (%)
0.5	0.569	1	0.237	0.864	0.227	0.021	9.40
		2	0.197	0.942			
		3	0.246	0.936			
0.6	0.683	1	0.363	0.947	0.361	0.024	6.57
		2	0.389	0.923			
		3	0.331	0.910			
0.7	0.796	1	0.472	0.926	0.465	0.025	5.46
		2	0.492	0.921			
		3	0.431	0.902			
0.8	0.910	1	0.562	0.925	0.587	0.042	7.21
		2	0.647	0.911			
		3	0.553	0.935			

## Data Availability

Some or all data, models, or codes generated or used during the study are available from the corresponding authors by request.
